# Analysis of nanopore detector measurements using Machine-Learning methods, with application to single-molecule kinetic analysis

**DOI:** 10.1186/1471-2105-8-S7-S12

**Published:** 2007-11-01

**Authors:** Matthew Landry, Stephen Winters-Hilt

**Affiliations:** 1 Department of Computer Science, University of New Orleans, New Orleans, LA, 70148, USA; 2 The Research Institute for Children, 200 Henry Clay Ave., New Orleans, LA 70118, USA

## Abstract

**Background:**

A nanopore detector has a nanometer-scale trans-membrane channel across which a potential difference is established, resulting in an ionic current through the channel in the pA-nA range. A distinctive channel current blockade signal is created as individually "captured" DNA molecules interact with the channel and modulate the channel's ionic current. The nanopore detector is sensitive enough that nearly identical DNA molecules can be classified with very high accuracy using machine learning techniques such as Hidden Markov Models (HMMs) and Support Vector Machines (SVMs).

**Results:**

A non-standard implementation of an HMM, emission inversion, is used for improved classification. Additional features are considered for the feature vector employed by the SVM for classification as well: The addition of a single feature representing spike density is shown to notably improve classification results. Another, much larger, feature set expansion was studied (2500 additional features instead of 1), deriving from including all the HMM's transition probabilities. The expanded features can introduce redundant, noisy information (as well as diagnostic information) into the current feature set, and thus degrade classification performance. A hybrid Adaptive Boosting approach was used for feature selection to alleviate this problem.

**Conclusion:**

The methods shown here, for more informed feature extraction, improve both classification and provide biologists and chemists with tools for obtaining a better understanding of the kinetic properties of molecules of interest.

## Background

Classification results are the ultimate judge of the success of whether a given feature or feature set is useful in the channel current-based signal analysis platform. Emission inversion and the addition of a spike density feature are shown to noticeably improve performance and are folded into a previously presented architecture [1]. It is also shown that Emission Variance Amplification (EVA) greatly reduces computation complexity and makes analysis of levels that are not well defined possible, while over-zealous use of tuning parameters can destroy kinetic information and thus render a channel current blockade signal useless. A new, efficient HMM-with-Duration is proposed as a solution [2-4]. Finally, although AdaBoost was not able to reproduce the best classification results obtained from a carefully selected feature set, AdaBoost is shown to be useful in several situations, including *ab initio *feature selection, and post feature selection pruning that offers similar results (not shown) to PCA-based feature selection on the same data (see [5], and references cited there, for a more comprehensive discussion of Adaboosting-based selection). Moreover, AdaBoost serves to validate the current, manually designed feature set.

### Nanopore detector

**Figure 1:**
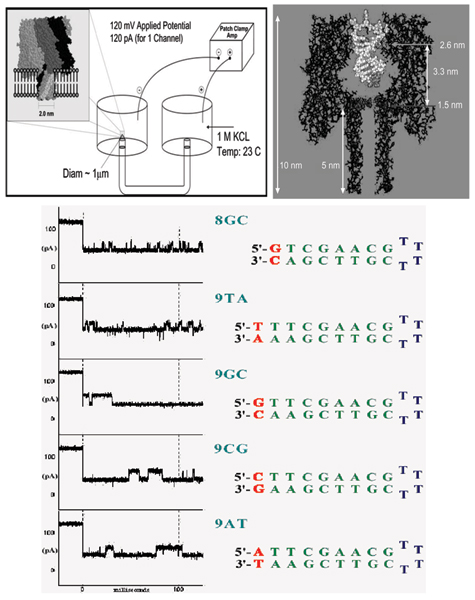
Left Panel: A lipid bilayer supports the alpha-hemolysin heptamer that creates a pore, or channel used to collect the data, as shown left. The channel is supported by an aperture, which allows the flow of ions between *cis *(here, left) and trans (here, right) wells. Right Panel: The assembled α-hemolysin pore shown to scale, with a captured dsDNA molecule. As shown, the double stranded form is too wide to pass through the pore, while a single strand may pass through. Bottom Panel: First 100 ms blockade patterns of four DNA hairpins, part of a test set of nine base-pair hairpins, with 4dT hairpin loops, that have been studied extensively, and an eight base-pair control. The nine base-pair molecules only differ in their terminal base-pairs, yet their channel current blockade signals, "signatures", are easily resolved [1].

The nanopore detector generates the data used in later stages of the channel current cheminformatics signal analysis architecture. A lipid bilayer supports the biologically-based channel. The channel used in what follows consists of a protein heptamer formed by protein monomers secreted by *Staphylococcus aureus*. Alpha-Hemolysin is used as the channel in the nanopore device due to its stable conformation (minimal gating) and its overall geometry (see Figure 1). The data consists of current reading through this channel. DNA and RNA interaction with the channel during translocation is non-negligible, but not strong enough for the molecule to get "stuck." Although dsDNA is too large to translocate, about ten base-pairs at one end can still be drawn into the large *cis*-side vestibule. This permits very sensitive experiments since the ends of "captured" dsDNA molecules can be observed for extensive periods of time to resolve features, allowing highly accurate classification of the captured end of dsDNA molecules [1,6-10]. In previous experiments, single molecules such as DNA have been examined in solution with nanometer-scale precision using nanopore blockade detection [1,6-8]. In early studies [8], it was found that complete base-pair dissociations of double stranded DNA to single stranded DNA could be observed for sufficiently short DNA hairpins. In later work [1,6], the nanopore detector was used to read the ends of double stranded DNA molecules and was operated as a chemical biosensor. In [6,9,10], the nanopore detector is used to observe the conformational kinetics of the end regions of individual DNA hairpins.

### Cheminformatics overview

**Figure 2:**
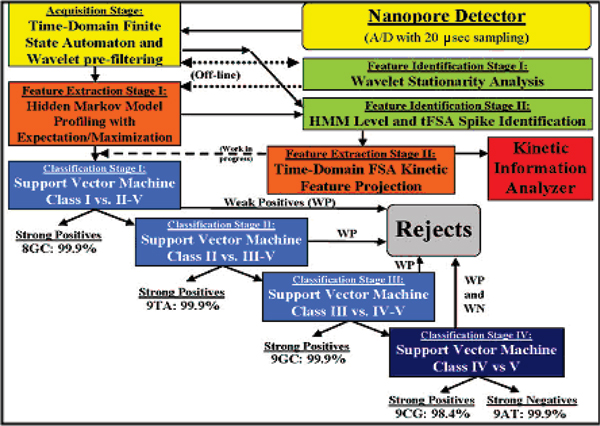
The channel current cheminformatics signal processing architecture.

The prototype channel current cheminformatics signal processing architecture "closes the loop" on the architecture previously presented in [1] (see Figure 2). The signal processing architecture is used to perform a preliminary test of pattern recognition informed (PRI) sampling control. As the nanopore detector generates data, a simplified time-domain Finite State Automaton (τFSA), shown in the figure in Additional File 1, is used for signal acquisition (see [4,11] for full model). The Bottom part of the figure in Additional File 1 describes the FSA that is used to find captures in a channel current data file. Only transitions between states are shown. Staying in the current state does not require any updating of the state of the FSA. Transitioning to another state requires only the recording of that sample index if the capture state is entered or exit. Note that only the current reading of the current observation and the current level count are needed to determine the state of the current observation. The current reading is used to determine the level and the current level count is used to ensure an actual level and not noise in the channel. The Top part of the figure shows a sample channel current blockade signal colored to correspond with the FSA. Once the signal is acquired, it is passed on to a generic HMM that is used to characterize current blockades and extract features [1-3,6]. During this step, the parameters of a generic-HMM are estimated using Expectation Maximization (EM) to effectively de-noise the signal [12]. After this stage, the extracted feature vector is passed on to an off-line-trained SVM. The classification result yielded by the SVM is then used to close the sampling control loop, i.e., undesirable molecules, or undesirable orientations of "capture", can promptly be ejected (by potential reversal). Further details on recent results on pattern-recognition-informed sampling control are presented in [3]. In this paper machine learning techniques and results, primarily in feature extraction and feature selection, are presented.

### HMM feature extraction

An HMM is used to de-noise and extract features from the acquired channel current signal. The HMM is implemented with fifty states. The only parameters necessary for determining a state is the current reading (which is given in picoamps) at a given point in the signal. This current reading is normalized to the baseline – taken to be the average current reading just before the capture event occurred. An average baseline reading of 120pA and a current reading of 70pA, for example, corresponds to a normalized value of 58.33% baseline. Then, using a bin size of one, the value of 58 is used as the current state. (Other bin sizes have been considered, but 1% granularity was sufficient for discerning structure, without burdening the HMM processing with too many states, and is used in what follows.) For most of the data studied in these experiments, almost all capture events take place between 20- and 70% blockade. Thus, only fifty states are used in an effort to help ease computational complexity – as input scales linearly, computation time scales quadratically. In the implementation of the HMM, the states are chosen with this observation in mind. In the previous example, our state of 58 would correspond to state 38. This process of scaling raw data to actual states is referred to as "quantization".

After the data is quantized, five rounds of Expectation Maximization are run to obtain accurate estimates of emission and transition probabilities. Initially, emissions for each state 'L', corresponding to a blockade at level 'L', is set to a gaussian with mean at L and unit variance. In addition, all transitions are equally likely. Expectation Maximization serves to obtain a more accurate measure of emissions and transitions based on the observed signal. A standard Viterbi algorithm is then run in order to de-noise the signal – that is, obtain the most likely path of states that created the observed signal. The process of finding the most likely path of states obtained by the Viterbi algorithm typically reduces the noise in the channel current signal.

After the Viterbi algorithm is run, a 150-component feature vector is created for the given signal. Each feature vector consists of three distinct sets of information. The first 50 components come directly from the 50 previously described states of the HMM. These components are level occupation probabilities (a histogram view) for each state calculated after the Viterbit trace back algorithm yields the most likely path. The second set of 50 components is composed of the variances of the emission probabilities. The third and final set of 50 components is composed of a weighted sum of transition probabilities from the dominant levels of a given signal.

One refinement to the standard implementation of an HMM, presented here, involves the initial manipulation of the emission probabilities as they are entered in to the HMM. The emission probabilities are the main place where the observed data is brought into the HMM-EM algorithm and can be viewed conceptually as the probability of emitting a hidden or true state given an actual or observed state. By exchanging the roles of the true and actual states, an additional contribution arises that is approximately a locally distributed entropy that is introduced at the cellular level in the standard Viterbi dynamic programming table (see Methods). While the exact theoretical underpinnings of this method are still being researched, it is clear that this "emission inversion" improves classification performance.

In addition to the 150-component feature vectors and the emission inversion technique already described, additional information can also be extracted. The effects of the addition of a spike density feature are explored, where a spike is defined as an anomalous, deep blockade of channel current from the lower level of a given signal.

Another variation on a standard HMM, Emission Variance Amplification is discussed. Here, the goal is to obtain dwell time information for the levels of a given molecule. From this information, the half-life, and thus, the stability of a given level can be determined. However, channel current data is noisy and building a Finite State Automaton to accurately model this noisy data can be difficult. Moreover, this model would not be easily re-usable for other channel current analysis without significant restructuring and re-tuning. Here, an HMM with EVA is used to reduce the gaussian noise bands around a given level while still strictly retaining transitions between levels. This method was first introduced in [2] and is used here to obtain the new results.

### AdaBoost

**Figure 3:**
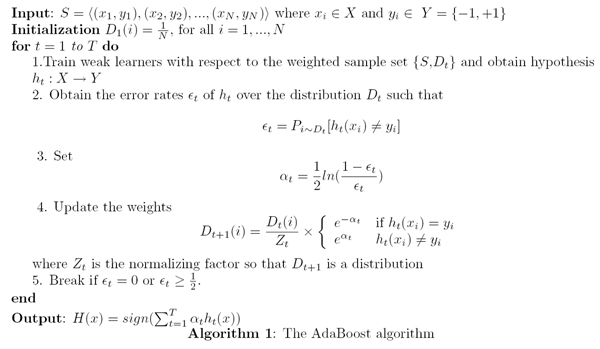
The traditional AdaBoost algorithm (graphic taken from [5]).

Adaptive Boosting (AdaBoosting) is typically used for classification purposes. In general, AdaBoost is an iterative process that uses a collection of weak learners to create a strong classifier. Training data is given a weight, and at each iteration, the weak learners are trained on this weighted data. Weights for these data points are then updated based on the error rate of the weak learner and whether a given data point was classified correctly or not. The consensus vote at each iteration is treated as a hypothesis, and weights are given to a hypothesis based on its accuracy. At the end of the iterative process, final classification is done using all hypotheses and their corresponding weights (see Figure 3). In this way, AdaBoost is able to use a set of weak learners to generate a strong classifier.

As a classification method, one of the main disadvantages of AdaBoost is that it is prone to over training. However, AdaBoost is a natural fit for feature selection. Here, over training is not a problem, as AdaBoost finds diagnostic features and those features are passed on to a classifier that does not suffer from over training such as a SVM. For this function, a modified form of AdaBoost is introduced.

### SVM classification

**Figure 4:**
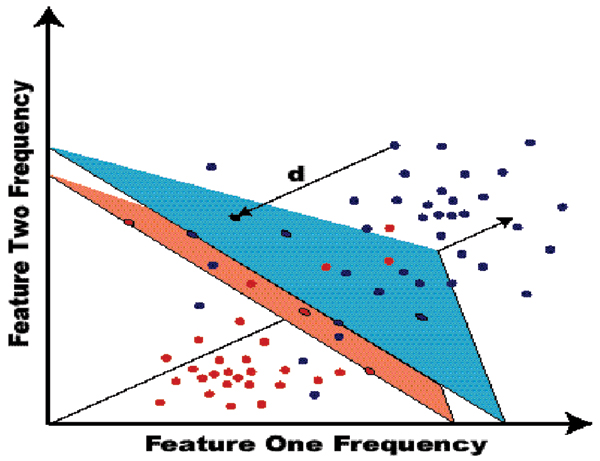
The hyperplane separability heuristic underlying the SVM classifier formulation, where the hyperplane is endowed with a thickness that is maximized (the SVM's structural risk minimization criterion).

Support Vector Machines (SVMs) are variational-calculus based methods that are constrained to have structural risk minimization (SRM) such that they provide noise tolerant solutions for pattern recognition [13,14]. Simply put, an SVM determines a hyperplane that optimally separates one class from another (see Figure 4). Once learned, the hyperplane allows data to be classified according to the region in which it resides.

The SVM approach encapsulates a significant amount of model-fitting information in its choice of kernel. In some sense, the SVM kernel provides a notion of distance to the decision hyperplane. Novel, information-theoretic, kernels were successfully employed for notably better performance over standard kernels in prior work[1,15].

Thus, SVMs are fast, easily trained, discriminators [13,14], for which strong discrimination is possible without the over-fitting complications common to neural net discriminators [13]. In these experiments, SVM classification performance is used as the benchmark for testing the validity of the various feature extraction permutations that are explored. This idea is a natural fit since one of the overarching goals of the nanopore detector is to be able to classify molecules based on their behavior in the channel. Furthermore, SVMs provide a natural confidence factor that can be leveraged when closing the sampling control loop.

## Results

In what follows, results are described for the proposed extensions and improvements to existing methods in the feature extraction architecture. Improvements in feature extraction and types of features are discussed. Specifically, emission inversion, the addition of a spike density feature, and HMM with EVA are discussed. In addition, a new method of feature selection is shown. The effects of using AdaBoost on a full set of transition probabilities versus a scheme for manually compressing transition probabilities are shown.

### Emission inversion

Observed data is brought into the HMM/EM process chiefly through the emission probabilities. Through running the HMM in debug mode and observing the interactions of various components, an interesting twist on traditional emission probabilities was found – when the observed states and emitted states share the same alphabet the roles of observed states and emitted states can be reversed for possible improvement to classification performance.

Data used from these experiments were the 9bphp data shown in Figure 1. For each of the binary classification problems considered, three different feature sets were chosen to analyze the effect of data inversion on SVM classification performance. The three sets selected for comparison were the manually designed 150-component feature vectors described in Background, the first set of 50 level occupation features from that 150-component set, and the second set of 50 variances on the emission probabilities from that 150-component set. The 9AT vs. 9TA, 9CG vs. 9TA, and 9GC vs. 9TA binary classification cases were selected to be shown here as they provide typical examples of the entire result set.

**Figure 5:**
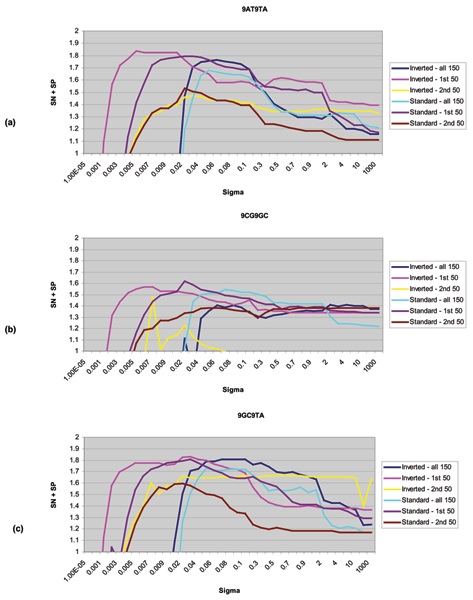
**(a) **SVM performance with different feature sets, for different binary classification data sets: 9AT vs 9TA. The Y-axis, "SN + SP", shows the sum of the Sensitivity and the Specificity. The X-axis is the kernel parameter σ. Note: standard ROC curves are not used here, and in what follows, for two reasons: (i) a comparison with common sigma parameterization was being explored, and (ii) SN and SP are not evaluated individually, whereas (SN + SP) is the measure employed for overall fitness evaluation of a given feature set, kernel, and kernel sigma. **(b) **SVM performance with 9CG vs 9TA. **(c) **SVM performance with 9GC vs 9TA. Throughout, the SVM shows that the feature set produced using the inverted emissions performs consistently better than the standard implementation of a HMM.

Experimentally, this emission inversion works well with channel current data as shown in Figure 5. These figures show SVM classification performance for the various feature sets just described using both a standard HMM implementation and a HMM implemented with data inversion as described here. The y-axis measures classification accuracy (sensitivity plus specificity) and the x-axis shows a tuning over the kernel parameter. The symmetric entropic kernel was used in this study as it has been shown to work well with channel current data in previous experiments [1]. The performance benefit is shown most notably in Figure 5a. In the case where the 150-component feature set was used, inverting the emissions yields a 5% peak increase in accuracy. This result is stable over a range of kernel parameter. For the case where the first 50 components were studied, a slight increase in classification performance as well as an increase in stability is observed. In Fig. 5c, a slight boost in classification performance is observed while a significant increase in stability is observed.

In nearly all cases studied, inverting the emissions provides a performance increase in accuracy, stability, or both accuracy and stability. For some molecules, this performance increase was more significant than others and in one case, out of the ten permutations studied, performance was marginally better using a standard HMM without emission inversion (but suffered from being less stable in its kernel parameter, so wouldn't be preferred anyway).

### Spike analysis

In addition to the level occupation probability, emission probability, and transition probability, the spike density from the lower level of a given molecule has been identified as a possibly significant feature. A spike event – an anomalous, deep blockade of channel current – from the lower level is conceptually seen as a fraying of the last few termini of a given molecule. Thus, a measure of spike density can yield information about the stability of the final few base pairings. For this analysis, data obtained from collaborators at NASA/AMES was used (see [16] for details). Here, the analysis is centered on two very similar 9GC molecules. On one of the molecules, the terminal guanine base was modified in an effort to simulate radiation damage. A blockade level histogram of the two signals (figure in Additional File 2) shows that there is high similarity between the blockades produced by the two molecules.

The spike detection method presented in [16] was used to identify spikes and extrapolate true spike counts as shown in the figures in Additional files 3 and 4. In those figures the blue curve represents actual spike counts observed versus a given cutoff. The red curve is drawn tangent to the observed curve. Thus, the true spike count is the reading as the tangent line crosses the x-axis. The molecule studied is a 9 base-pair hairpin that is the radiation damaged DNA model (a terminal guanine is oxolated) (see [16] for details), with terminal guanine unaltered in the "non-radiated" molecule. The spike count plots show increasing counts as spike cut-off thresholds are relaxed (to where eventually any downward deflection will be counted as a spike). The linear phases of spike count increase, with threshold relaxation, is associated with instances of anomalous "spike noise" and forms the basis for a heuristic for defining the spike feature. Plots are automatically generated using gnuplot and automatically fit with extrapolations of their linear phases at the group's tools website. The extrapolations provide an estimate of "true" anomalous spike counts – counts associated with terminus fraying in the captured DNA hairpin (as shown in [7]). The radiated form of the molecule frayed 17.6 times on average (while in the LL state), and is shown in Additional File 3, while the non-radiated molecule only frayed 3.58 times a second, on average, while in its lower-level state (Additional File 4).

**Figure 6:**
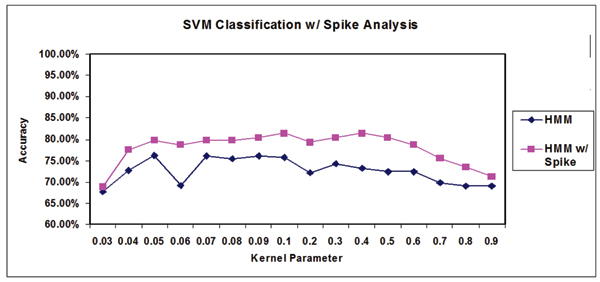
Example classification results with and without spike analysis. Note that adding a spike feature significantly improves classification accuracy over a wide range of kernel parameters. The Y-axis, "Accuracy", is taken to be (SN+SP)/2 expressed as a percentage, to be consistent with the prior SN+SP based measure, so is not the conventional accuracy = (TP+TN)/(TP+TN+FP+FN). The X-axis is the kernel parameter σ.

Building on the efforts in [16], this spike density feature was used as a single feature and concatenated to the end of the 150-component feature vector (described in Background). The results of this analysis are shown in Figure 6 (similar to the description of the emission inversion results in the previous section). Incorporation of this spike feature for this data set leads to classification with approximately 5% greater accuracy over a wide range of tuning parameters. It is noteworthy that the addition of only one extra feature, the spike density feature, yields a significant performance increase.

### Dwell time analysis using emission variance amplification

Another important feature of a channel current blockade signal is the duration of blockade levels. However, acquiring level duration information is a non-trivial task due to a significant gaussian noise band around blockade levels. The goal here is to use Emission Variance Amplification in the HMM with EM to drastically reduce noise in the signal while still precisely retaining level transitions. By retaining the level transitions, the integrity of the kinetic information – level dwell times in this case – remain in tact.

Data used for this analysis was gathered from a simple study of DNA-DNA annealing using the nanopore detector and a Y-aptamer transduction platform. Results on blockade states observed for Y-aptamer overhang+complement binding study are shown in the figures in Additional Files 5 and 6. Additional File 5 shows the 150-component feature vector profiles for the Y-aptamer that binds a 6A ssDNA, for signals before and after introduction of that six adenosine ssDNA (from [18]). Additional File 6 shows the dwell time distributions for the three dominant levels of the Y-aptamer (without 6A target). For further details and Results, see the work presented in [18] in this same Journal.

**Figure 7:**
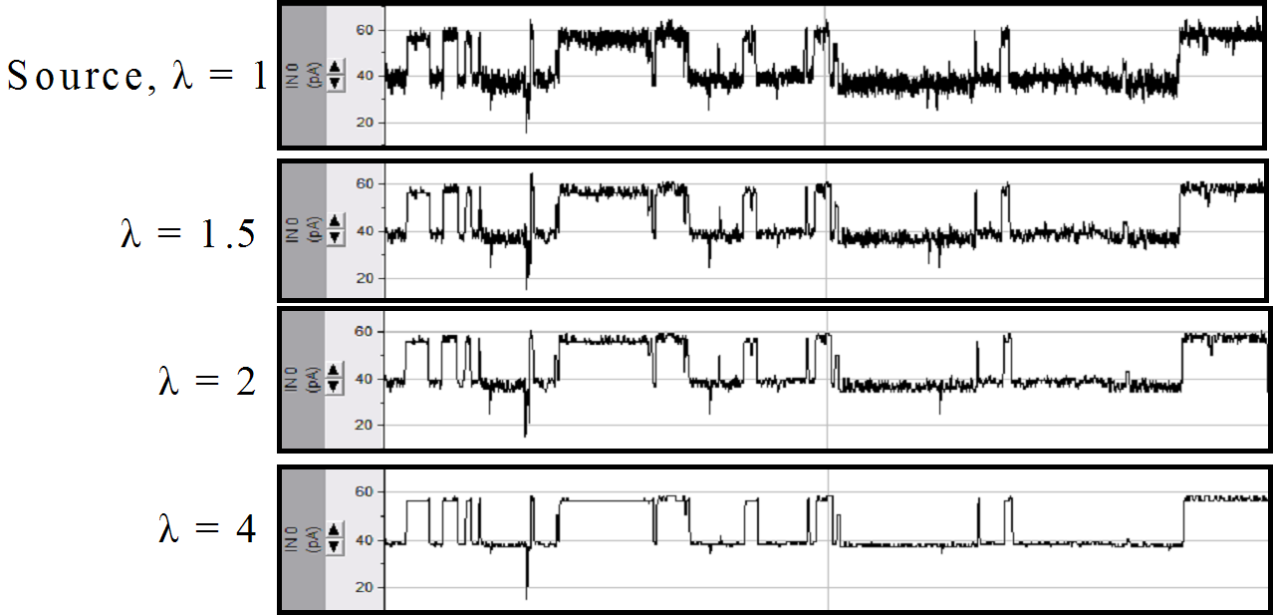
As the EVA factor increases, the gaussian noise surrounding the levels is reduced significantly, yet level transitions are strictly retained.

Visually, the results of EVA can be seen in Figure 7. Note that as the variance is amplified from the original setting of 1, the noise band around a given level is reduced significantly. Moreover, even though many spike events are destroyed, transitions between dominant levels – and thus level dwell times – are strongly retained. After EVA pre-processing, a trivial Finite State Automaton can now extract dwell time information. This FSA only needs a current reading and a duration (in sample counts) to characterize any given level. Without EVA, a wide range of current cutoffs or even some more complex model would be needed to characterize a given level. But, using this simplified FSA, dwell time distributions for the studied data were easily obtained (see the figure in Additional File 6). From these dwell time distributions, the half-life – and thus a measure of level stability – can be gathered. This half-life is an important kinetic characteristic for a biologist or chemist studying the properties of a molecule. Future work will evaluate whether half-lives of levels or even entire dwell time distributions can be useful in improving classification performance.

### Feature selection with AdaBoost

As has been shown in the spike analysis, careful selection of features plays a significant role in classification performance. However, adding non-characteristic or noisy features will hurt classification performance. In addition, recall from the discussion in Background that the last set of 50 components from the baseline 150-component feature vector are compressed transition probabilities. With a 50 state HMM, there would be 50*50 or 2500 possible transitions. However, a means of compression is necessary because many of these transitions are very unlikely and contribute noise to the feature vector. Without compression, classification performance suffers as a result, yet it is uncertain as to whether diagnostic information has been inadvertently discarded in the manual compression of the transition probabilities. An automated approach is desired to solve the issue of feature selection. Here, a hybrid AdaBoost approach is used as an automated, objective means of feature selection.

The data studied for feature selection include the 9CG vs 9GC and 9GC vs 9TA binary classification problems from the 9bphp data used in the data inversion analysis (Figure 1). The 9GC vs 9TA set was studied first. Since the 9GC vs 9TA case is one of the easier classification problems with this dataset, the 9CG vs 9GC case was also analyzed. This case is among the hardest binary classification problems in this dataset.

**Figure 8:**
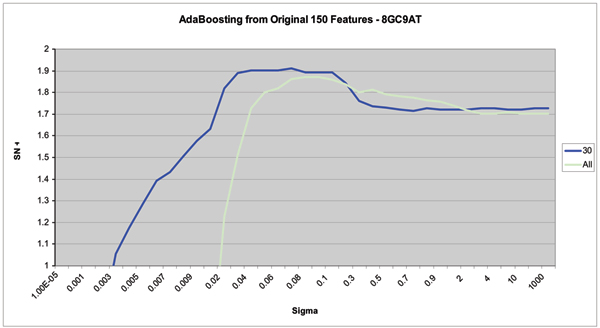
If Adaboost operates from the 150-component manual set, a reduced feature set of 30 is found to work best, and with notable improvement in kernel parameter stability in the region of interest. (The Y-axis, "SN + SP", shows the sum of the Sensitivity and the Specificity. The X-axis is the kernel parameter σ.)

**Figure 9:**
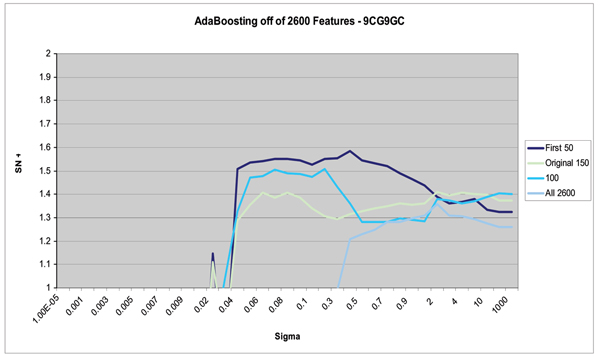
AdaBoosting to select 100 from the full set of 2600 features improves classification over just passing all 2600 components to the SVM. However, the best performance is still obtained when working with the Adaboosting from the manual set. (The Y-axis, "SN + SP", shows the sum of the Sensitivity and the Specificity. The X-axis is the kernel parameter σ.)

**Figure 10:**
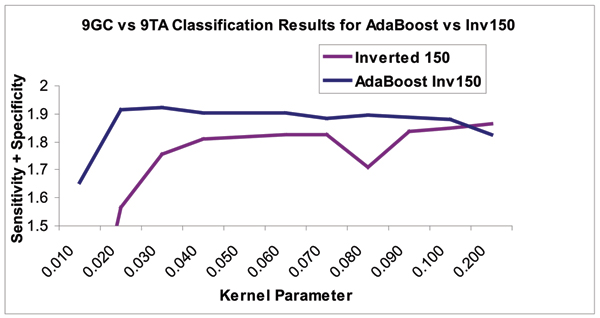
Classification improvement with Adaboost taking the best 50 from the Inverted-emission 150 feature set. 95% accuracy is possible for discriminating 9GC from 9TA hairpins with no data dropped with use of Adaboost, without Adaboosting, the accuracy is approx. 91%. This demonstrates a significant robustness to what the SVM can "learn" in the presence of noise (some of the 2600 component have richer information, but even more are noise contributors). This also validates the effectiveness with which the 150-parameter compression was able to describe the two-state dominant blockade data found for the nine base-pair hairpin and other types of "toggler" blockades, as well as the utility of the inverted features.

Figures 8, 9, 10 show the results of this automated feature selection analysis (these figures have a similar description to the figures described in the Emission Inversion results section). Figure 9 shows the effects of AdaBoosting off of the full, uncompressed feature vectors. These feature vectors are comprised of the 50 blockade level components (same as from the 150-component set), the 50 variances on the emission probabilities (same as from the 150-component set), and the full 2500 transition probabilities. Using a SVM to classify all 2600 features shows a notable decrease in classification accuracy and a significant decrease in the stability of classification results. AdaBoost is used to select the top 100 diagnostic features. These 100 features are extracted from the full 2600-component set of features and passed on to the SVM for classification. In this case, classification outperforms both the full 2600-component set and the manually designed 150-component set. The curve denoted by "First 50" represents the first 50 blockade level probabilities. This set is the best performing manually designed set, and outperforms the AdaBoost selected feature set in both performance and stability. Figure 10 shows the results of AdaBoosting off of the manually designed 150-component feature set in the case of the 9GC vs 9TA binary classification problem. There is a notable performance increase in classification accuracy and stability.

## Discussion

In what follows, the pros and cons of each proposed method presented in the Background and Results sections are discussed. In addition, proposed fixes and future work is discussed.

### Emission inversion

Emission inversion involves exchanging of the roles of emitted states and observed states. The exact theoretical underpinning of exchanging these roles is not yet completely understood (see Methods for details). In some sense, however, classification performance is the ultimate judge of the validity of a given method. As described in the Results section, the SVM classification performance is strongest when using emission inversion.

There are currently a couple of caveats. Emission inversion only works when the emitted and observed states share the same alphabet – with the channel current blockade analysis platform this restriction holds. Another caveat is that this method may be strongly data dependent. Only channel current data has been studied using this method for feature extraction, and it is entirely possible that emission inversion does not improve classification on other datasets. In this particular application, the AdaBoost feature selection provides a simple fix to the choice of what features to use. Simply create datasets that include extracted features from both a standard HMM implementation and a HMM implementation with emission inversion and let AdaBoost select the most diagnostic features in an automated way.

### Spike analysis

The results described above clearly show that spike density from the lower level is an important feature. Obtaining the spike density feature (described in Methods) is straightforward. However, adding this feature to the existing 150- or 2600-component feature sets currently requires tuning. Simply adding the spike density feature to an existing feature vector already containing 150 features will obscure the effect of the spike density feature almost completely. Thus, a weight must be added to this new feature. Should the weight be too heavy, though, the effect of the other features will be obscured. Currently, the weighting factor is tuned over in order to arrive at a weighting such that the spike density feature improves classification without obscuring the contribution of other features.

A few automated solutions are suggested for future work. One proposed solution is to simply add the un-weighted spike density feature to the existing feature vector and use AdaBoost to select the most diagnostic features. This approach will essentially create a weight for the spike density feature. That is, by removing many components that only add noise to a given feature vector, the remaining features are given more weight. Another solution that is currently being worked on is to fold the definition of a spike into the HMM. This solution requires a non-trivial amount of work as the entire definition of a state has to be entirely reworked. Moreover, the definition of a state must be considered carefully such that a state explosion (as seen in higher order HMMs) does not occur.

### Dwell time analysis

Preprocessing channel current blockade data using a HMM with EVA significantly reduces the complexity of dwell time analysis. Within a reasonable range of values for EVA factor, the noise bands around levels are significantly reduced while level transitions are retained. However, if too large of an EVA factor is used then transitions can be destroyed and the channel current signal will be mangled beyond use. Although this problem is not significant for a wide range of EVA factor, a HMM with Duration [2-4] will retain transitions and can eliminate this problem altogether.

Another aspect of the dwell time analysis that will be explored in future work is the effect of dwell time information on classification. Dwell time distributions for dominant levels should be characteristic for a given signal and thus improve classification performance. However, a significant amount of data is generally necessary to generate accurate dwell time distributions. In the current architecture, 100 ms of channel current blockade are analyzed to create one feature vector. It is unclear as to whether 100 ms will be enough data to overcome this limitation on sparseness of data. A longer signal trace could be analyzed, but computational complexity grows quadratically as signal input increases linearly. Here, the use of a distributed HMM has been developed to allow for the processing of enough data to provide accurate dwell time statistics while still meeting reasonable time constraints (paper in preparation), using a simple distribution analogous to the chunk processing that is employed for the SVM training [17].

### Feature selection

Typically AdaBoost is used as a classification method. But due to the limitations discussed in the Background section, SVMs provide a much more robust means of classification for channel current data. However, AdaBoost is still useful in feature selection, and that is the main use we have for AdaBoost in the work presented here. The weighting schemes in the AdaBoost algorithm are a natural fit for feature selection as the weights indicate which features are most diagnostic for a given classification problem.

AdaBoost does require some subtle tuning. As can be seen in the algorithm shown in Figure 3, AdaBoost does not have a natural end point. Unlike an SVM, AdaBoost does not converge on a solution. The number of iterations in the AdaBoosting algorithm must be tuned over in order to ensure accurate results. Another tuning parameter is the number of diagnostic features "D" to select from the original feature set "O". Should D be chosen too small, diagnostic features existing in O will be excluded and classification performance will suffer. Should D be chosen too large, noisy features existing in O will exist in D and classification performance will suffer. In general, though, the choice of D does not present a great problem as SVMs are robust and can learn well in the presence of noise and non-diagnostic features. Experimentally it has been observed that it is more important that D not be chosen too small as opposed to too large.

It is also important to note that automated feature selection using AdaBoosting was not able to reproduce results obtained from the "best-case" manually designed feature set (see Figure 9). Nonetheless, feature selection using AdaBoost is an important technique. It allows for the automated exploration of the effect of many different features and feature sets. In addition, AdaBoosted feature selection would be useful in problems where the definition of states do not lead to an easily designed manual set of features.

## Conclusion

Several new techniques and improvements on existing techniques in the channel current signal analysis platform have been introduced. Data inversion was introduced and was shown to be an improvement over the standard implementation of a HMM in regards to channel current data and final classification performance. Previous methods for spike feature extraction were folded into the current architecture. In addition, a new method for analyzing dwell times, Emission Variance Amplification was applied to the HMM. Finally, a hybrid AdaBoost approach was introduced in an effort to improve the feature selection process. Not only are these techniques useful improvements for the current signal process architecture, but several techniques introduced here also provide means to move forward with future research as detailed in the Discussion section.

## Methods

### Emission inversion

As previously discussed in the Background and Discussion sections, the main place where data is introduced into the HMM/EM algorithm is through the emission probabilities. In the HMM, emissions are defined as a multidimensional array and can be viewed conceptually as the probability of a hidden state emitting an observed state:

emission_probabilities [state] [observed_value] ≡ P(X = b|S = k),

where b = observed_value and k = state. A standard implementation of a HMM would be implemented in the following manner:

   For (I = 0; I < NUM_STATES; I++) {

      Forward [0] [I] = emission_probabilities [I] [observed_data[0]] *

         Prior_probability [I];

   }

The data inversion implementation simply exchanges the roles of the actual state and the observed state as follows:

   For (I = 0; I < NUM_STATES; I++) {

      Forward [0] [I] = emission_probabilities [observed_data[0]] [I] *

         Prior_probability [I];

   }

This simple inversion introduces another information factor into the Viterbi algorithm and improves performance as discussed in the Results section. So, with inversion, instead of P(X = b|S = k) we now have P(X = k|S = b). In our analysis we have P(X = k|S = b) ≈ P(S = k|X = b), so the change with inversion is approximately a factor of [P(S = k)/P(X = b)] introduced at each column position. For the Viterbi calculation, with sums on log contributions from each column, i.e., log [P(S = k)/P(X = b)], the new term sums to the length-weighted relative entropy between the state prior probability and emission posterior probability: - L D(X||S), where L is the length of data parsed and 'D(* || *)' is the Kullback-Leibler Divergence (or relative entropy).

### Emission variance amplification

As mentioned in the Discussion section, a HMM with EVA is used to significantly reduce the gaussian noise band around levels. In a non-EVA approach, emission probabilities are initialized with a gaussian profile. The initialization is as follows:

emission_probabilities [i] [k] = exp(-(k-i)*(k-i)/(2*variance))

where "i" and "k" are each a state with 0 <= {i, k} <= 49 in a 50 state system. To perform EVA, the variance is simply multiplied by a factor that essentially widens the gaussian distribution imposed on possible emissions, and the equation simply becomes

exp(-(k-i)*(k-i)/(2*variance*eva_factor)).

Essentially EVA boosts the variance of the distribution and yields the following effect: for states near a dominant level in the blockade signal, the transitions are highly favored to points nearer that dominant level. This is a simple statistical effect having to do with the fact that far more points of departure are seen in the direction of the nearby dominant level than in the opposite direction. When in the local gaussian tail of sample distribution around the dominant level, the effect of transitions towards the dominant level over those away from the dominant level can be very strong. In short, a given point is much more likely to transition towards the dominant level than away from it.

### Feature selection

As introduced in the Backgrounds and Discussion sections, AdaBoost is used in feature selection. In this hybrid implementation, weights are given to the weak learners as well as the training data. The key modifications here are to give each column of features in a training set a weak learner and to update each weak learner every iteration, not just updates the weights on the data. Conceptually, this idea can be seen in the figure shown in Additional File 7. Training data can be viewed as a two dimensional array of feature components. F_1 _- F_j _are individual feature vectors representing a single capture event. E_1 _- E_i _are the experts or weak learners assigned to an individual component in feature space. In the implementation described in this paper, naïve bayes classifiers were used as weak learners.

For a given number of iterations T, the process is as follows:

   Initialize weights on weak learners

   Initialize weights on training data

   For i, 1..T

      Train weak learners

      Update the weights for each weak learner – just like the hypothesis

         update in the standard AdaBoosting method

      Update the weights for each training point – just like the original

         AdaBoosting method

      Normalize the two weights.

      Break if the overall composite learner's error rate is 0% or 50%

In an example where there is a set of 150-component feature vectors, 150 weak learners would be created. As previously mentioned, each weak learner corresponds to a single component and classifies a given feature vector based solely on that one component. Then, weights for these weak learners are introduced. In each iteration of this modified AdaBoost process, weights for both the input data and the weak learners are updated. The weights for the input data are updated as in the standard AdaBoost implementation while weights on the individual weak learners are updated as if each were a complete hypothesis in the standard AdaBoost implementation (see figure in Additional File 7). At the end of the iterative process, the weak learners with the highest weights, that is, the weak learners that represent the most diagnostic features, are selected and those features are passed on to a SVM for classification. Thus, the benefits of both AdaBoost and SVMs are obtained.

## Competing interests

The authors declare that they have no competing interests.

## Authors' contributions

The initial submission was written by ML and SWH, with revisions by SWH. The core feature extraction and pattern recognition software was developed by SWH. The AdaBoost refinement and test dataruns were done by ML.

## Supplementary Material

Additional file 1 A simplified time-domain Finite State Automaton (τFSA) is used for signal acquisition (see [4,11] for full model). Click here for file

Additional file 2 A blockade level histogram of two DNA hairpin channel blockade signals. Click here for file

Additional file 3 The extrapolated true spike counts for the radiated DNA hairpin blockade. Click here for file

Additional file 4 The extrapolated true spike counts for the non-radiated DNA hairpin blockade. Click here for file

Additional file 5 The 150-component feature vector profiles for the Y-aptamer that binds 6A ssDNA, for signals before and after introduction of that six-adenosine ssDNA. Click here for file

Additional file 6 The dwell time distributions for the three dominant levels of the Y-aptamer (without 6A target). Click here for file

Additional file 7 The key Adaboost modifications are to give each column of features in a training set a weak learner, and to update each weak learner every iteration, not just update the weights on the data. Click here for file
