# Supplementary figures and images for: Analysis of nanopore detector measurements using Machine-Learning methods, with application to single-molecule kinetic analysis
Source: BMC Bioinformatics. 2007 Nov 1;8(Suppl 7):S12. doi: 10.1186/1471-2105-8-S7-S12 (PMC2099480; doi:10.1186/1471-2105-8-S7-S12)

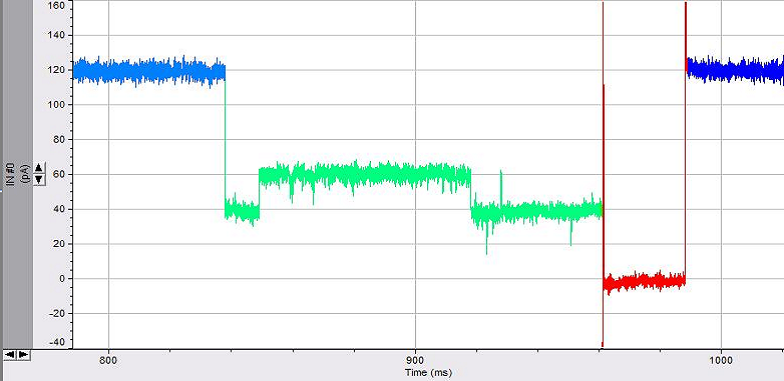

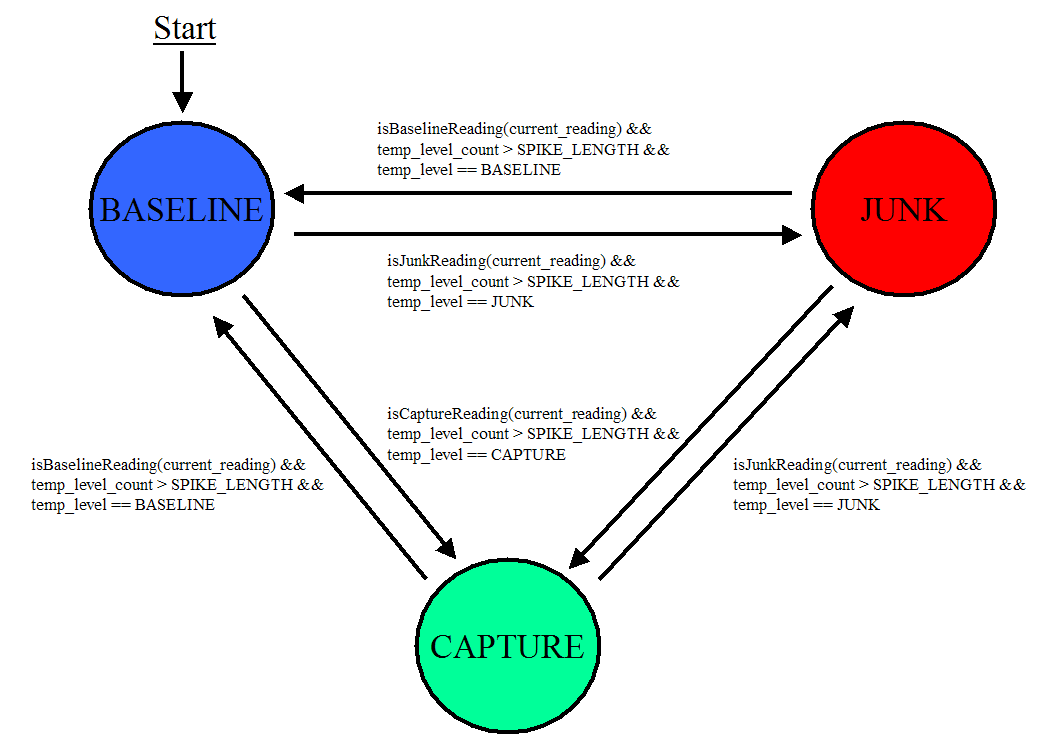

Supplement: Additional file 1 — A simplified time-domain Finite State Automaton (τFSA) is used for signal acquisition (see [4,11] for full model). [file 1471-2105-8-S7-S12-S1.doc]

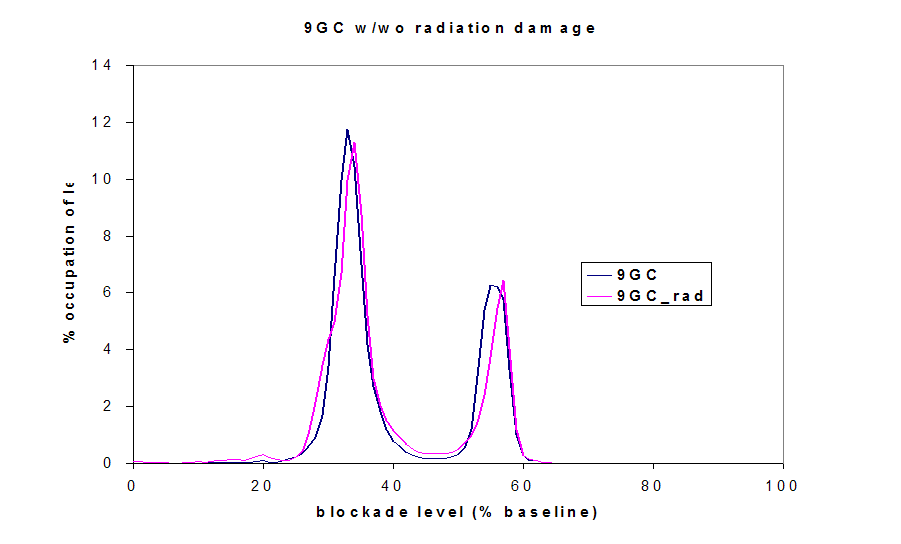

Supplement: Additional file 2 — A blockade level histogram of two DNA hairpin channel blockade signals. [file 1471-2105-8-S7-S12-S2.doc]

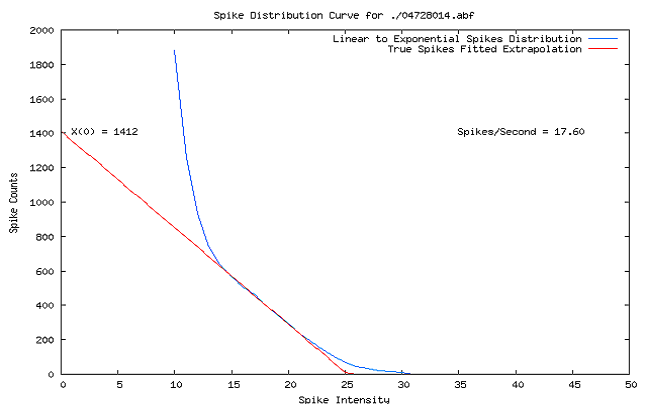

Supplement: Additional file 3 — The extrapolated true spike counts for the radiated DNA hairpin blockade. [file 1471-2105-8-S7-S12-S3.doc]

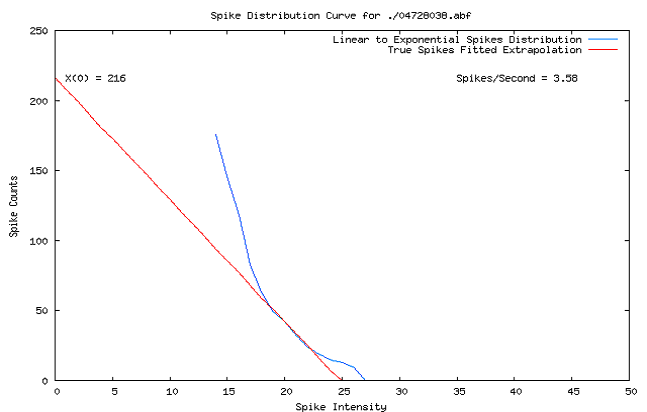

Supplement: Additional file 4 — The extrapolated true spike counts for the non-radiated DNA hairpin blockade. [file 1471-2105-8-S7-S12-S4.doc]

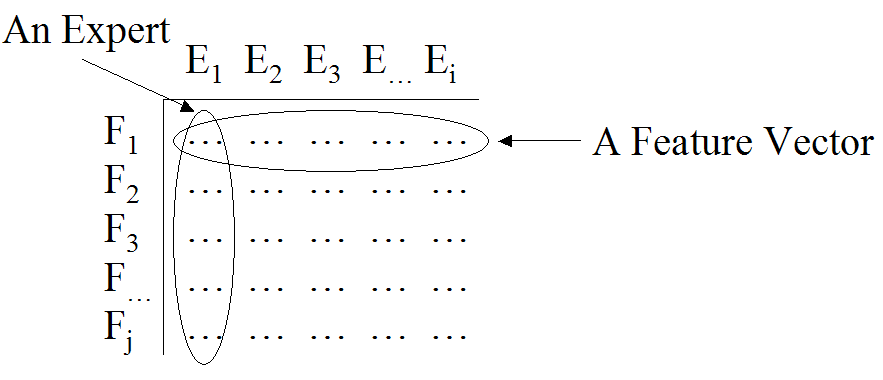

Supplement: Additional file 7 — The key Adaboost modifications are to give each column of features in a training set a weak learner, and to update each weak learner every iteration, not just update the weights on the data. [file 1471-2105-8-S7-S12-S7.doc]
