# Supplementary material for: Analysis of nanopore detector measurements using Machine-Learning methods, with application to single-molecule kinetic analysis
Source: BMC Bioinformatics. 2007 Nov 1;8(Suppl 7):S12. doi: 10.1186/1471-2105-8-S7-S12 (PMC2099480; doi:10.1186/1471-2105-8-S7-S12)
Supplement: Supplementary file 2 [file 1471-2105-8-S7-S12-S5.doc]

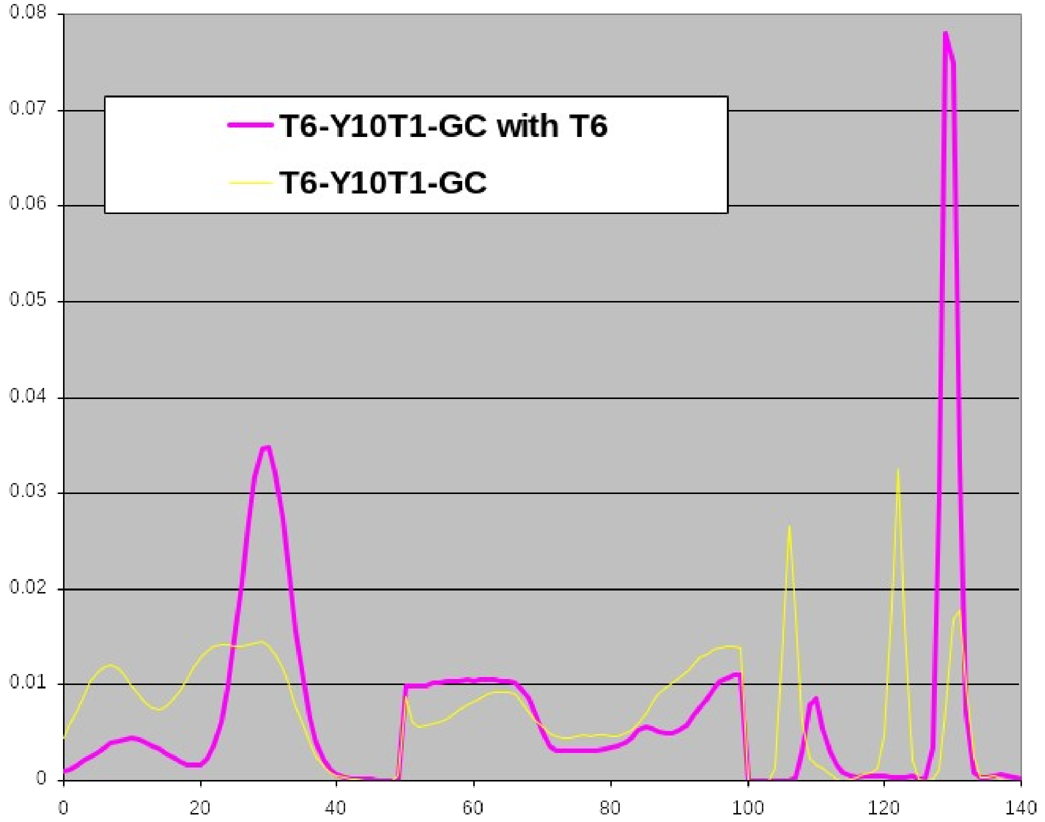

T6-Y10T1-GC with T6
T6-Y10T1-GC
0.08
0.07
0.06
0.05
0.04
0.03
0.02
0.01
0
0
20
40
60
80
100
120
140
