# Supplementary material for: Analysis of nanopore detector measurements using Machine-Learning methods, with application to single-molecule kinetic analysis
Source: BMC Bioinformatics. 2007 Nov 1;8(Suppl 7):S12. doi: 10.1186/1471-2105-8-S7-S12 (PMC2099480; doi:10.1186/1471-2105-8-S7-S12)
Supplement: Supplementary file 3 [file 1471-2105-8-S7-S12-S6.doc]

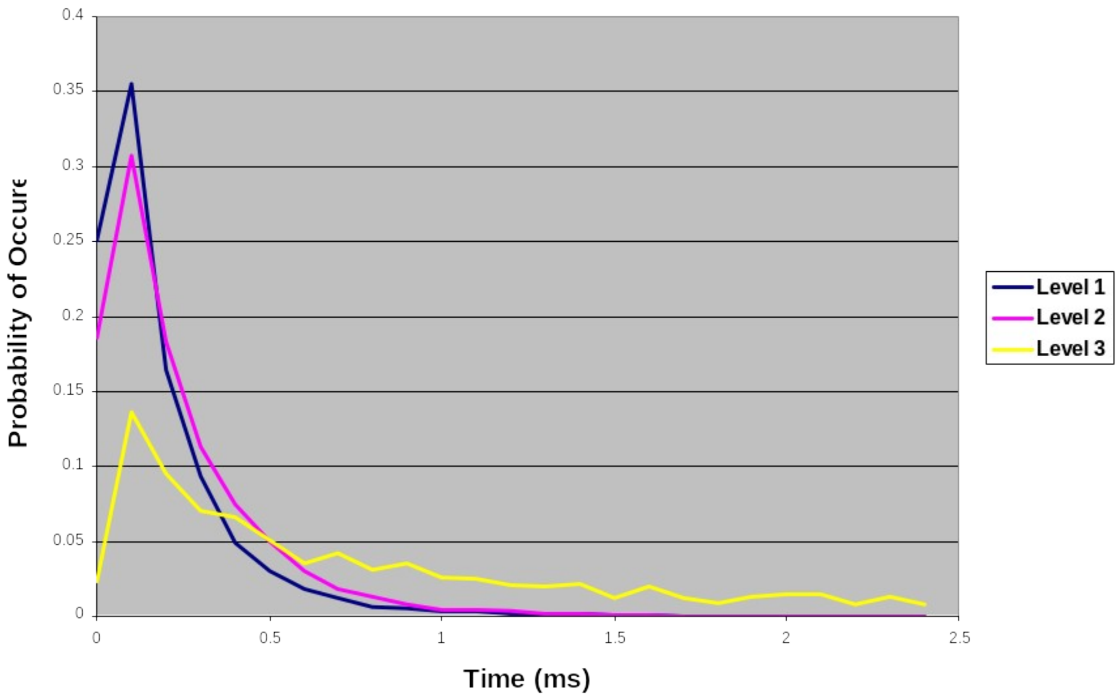

Probability of Occure
Time (ms)
Level 1
Level 2
Level 3
0
0.05
0.1
0.15
0.2
0.25
0.3
0.35
0.4
0
0.5
1
1.5
2
2.5
